# Performance of Diffusion Monte Carlo Calculations for Predicting the Relative Energies of Quinoidal and Nonquinoidal Species

**DOI:** 10.1021/acs.jpclett.5c03120

**Published:** 2025-12-12

**Authors:** N. Mauger, A. Benali, K. D. Jordan

**Affiliations:** † Department of Chemistry, 6614 University of Pittsburgh, Pittsburgh, Pennsylvania 15218, United States; ‡ Computational Science Division, Argonne National Laboratory, Lemont, Illinois 60439, United States

## Abstract

Coupled cluster singles and doubles with perturbative triples [CCSD­(T)] and single determinant fixed-node diffusion Monte Carlo (SD-DMC) have emerged as two of the most useful methods for providing benchmark reaction and interaction energies of chemical systems without strong static correlation. The errors in DMC energies are dominated by an inexact description of the nodal surfaces for electron exchange. One of the main approaches to addressing the fixed-node error is to use multideterminant (MD) trial wave functions. We consider here the energy differences between pairs of related molecules with aromatic and quinoidal structures as well as between quinoidal isomers. Quinoidal systems tend to have some diradical character, leading one to anticipate that SD-DMC calculations may face challenges in accurately describing their energetics. The MD trial wave functions were generated from the complete active space calculations. A comparison is made with the predictions of well-converged CCSD­(T) calculations.

## Graphical abstract

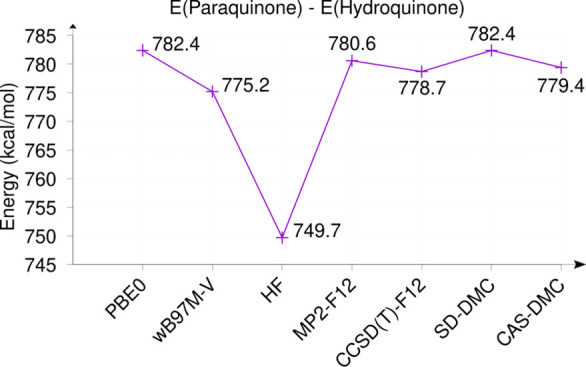

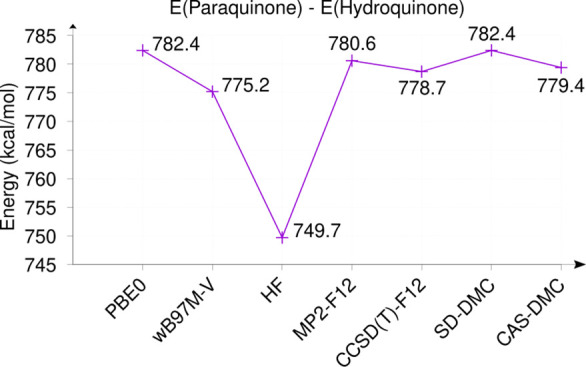

Significant effort has been dedicated to obtaining accurate benchmark results for atomization energies, noncovalent interaction energies, and reaction energies.^1–6^ Such data are essential for the development and validation of new electronic structure methods, for parametrizing force fields, and for training and testing machine learning models.^7,8^ For many purposes, the target of benchmark quantum chemical calculations is 1 kcal/mol, which is often taken as the threshold for “chemical accuracy”. In general, even the most sophisticated density functional theory (DFT) methods failed to achieve this target. For systems for which the Hartree–Fock (HF) method provides a good starting wave function, chemical accuracy can often be achieved with the coupled cluster singles plus doubles with perturbative triples [CCSD­(T)] ^9,10^ method, provided sufficiently flexible basis sets or extrapolation to the complete basis set (CBS) limit are employed. Although the steep computational scaling of CCSD­(T) limits it to relatively small systems, with the use of localized orbital approximations as in the domain-based local pair natural orbital coupled-cluster [DLPNO-CCSD­(T)] ^11,12^ and pair-natural orbital local coupled cluster [PNO-LCCSD­(T)] ^13,14^ methods, the scaling with system size can be greatly reduced. However, there remains the need to use large basis sets to achieve chemical accuracy. Increasingly, diffusion Monte Carlo (DMC)^15–17^ calculations are being used to provide benchmark electronic structure data.^18–21^ The DMC method has the advantages of displaying relatively low (*N* ^2^ – *N* ^4^) scaling with the number of electrons, *N*, being much less sensitive to the basis set than traditional wave-function-based methods and being highly parallelizable.

To ensure Fermionic behavior when applied to electronic structure problems, DMC calculations generally make use of the fixed-node approximation^22,23^ in which the nodal surface for exchange of electrons is imposed by use of a trial wave function. Most commonly, the trial wave function is taken to be a single Slater determinant (SD) of HF or DFT orbitals multiplied by a Jastrow factor.^24,25^ The Jastrow factor is symmetric with respect to electron exchange and, thus, does not impact the nodal surface. SD-DMC performs well for many systems but can have significant energy errors in systems with a strong static correlation. One of the most common approaches to reducing the fixed-node error is the adoption of multideterminant (MD) trial wave functions.^26–30^ However, the relationship between fixed-node errors and the underlying multiconfigurational nature of the system remains poorly understood.

One might expect that accurate prediction of relative energies between molecules with quinoidal and those with nonquinoidal structures to present a challenge for SD-DMC due to the partial diradical character associated with quinoidal systems. Systems with appreciable diradical character can have strong static correlation, which impacts the nodal surface and, consequently, the accuracy of DMC energies. Even moderate diradical character can lead to substantial fixed-node errors. This sensitivity has been explored in previous work,^31–34^ including a study by one of the authors,^35^ where a geometric change in the rectangular H_4_ system increasing the weight of the second most important configuration in a complete active space self-consistent-field (CASSCF) wave function from 0.14 to 0.21 resulted in over a 10-fold change in the energy difference between SD- and MD-DMC calculations, underscoring the significant impact of diradical character on fixed-node errors.

Building on this, we consider the energy differences between paraquinone (PQ) and hydroquinone (HQ), between the three benzoquinone isomers, between *p*-xylylene and *p*-xylene, and between two protomers of 4-aminobenzoic acid (4ABA), using DMC with both SD and MD trial wave functions, as well as several other electronic structure methods including CCSD­(T). Protonated 4ABA can exist as either the O-protomer (carboxylic group) or the N-protomer (amine group). PQ, *p*-xylylene, and the O-protomer of 4ABA exhibit quinoidal structures, which are characterized by significant bond-length alternation in the ring (see Figure 1). However, we expect PQ and *p*-xylylene to exhibit greater diradical character and to prove more problematic for SD-DMC than the O-protomer of 4ABA. Of the three benzoquinone isomers, that with the carbonyl groups in the 1 and 3 ring positions is known to have the greatest diradical character.^36,37^

**Figure 1:**
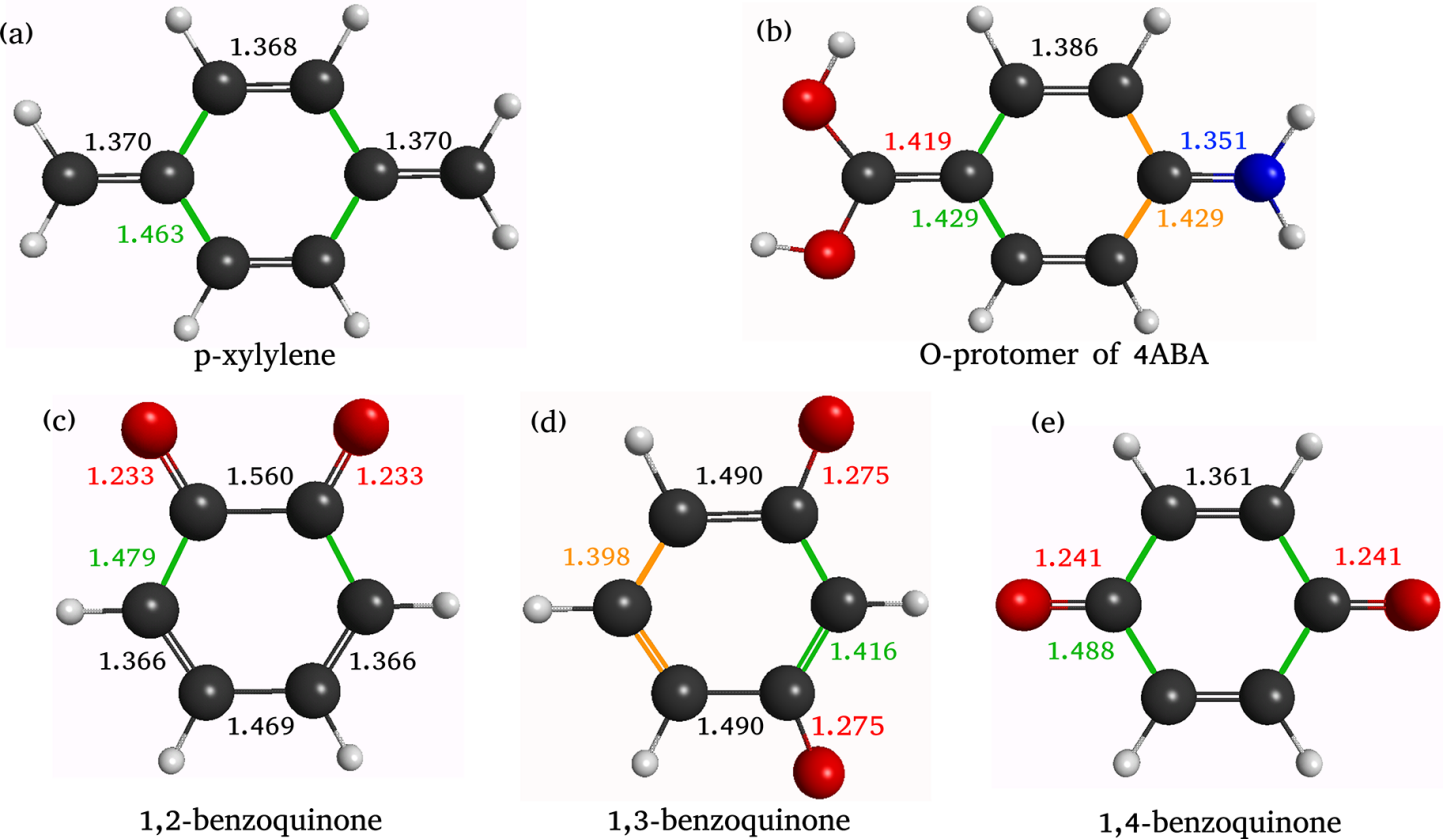
Chemical structures of (a) *p*-xylylene, (b) the O-protomer of 4ABA, (c) 1,2-benzoquinone, (d) 1,3-benzoquinone and (d) 1,4-benzoquinone. Bond lengths in Å calculated at the MP2/aug-cc-pVDZ level of theory are indicated.

The geometries of the eight molecules of interest were optimized using second-order Møller–Plesset perturbation theory (MP2)^38^ using the aug-cc-pVDZ basis set^39,40^ and the frozen-core approximation. The geometries are reported in the SI. The trans isomer was used for HQ, while the EZ isomer was employed for the O protomer of 4ABA. These geometries were used for single-point calculations carried out using the SD-DMC, MD-DMC, HF, and CCSD­(T) methods as well as using the PBE0 ^41,42^ and ωB97M-V^43^ density functionals. PBE0 is a hybrid functional, while ωB97M-V is a range-separated meta generalized gradient approximation functional GGA. The single-point MP2 and the majority of the single-point CCSD­(T) calculations used the frozen-core approximation and were combined with the explicitly correlated F12-c procedure.^44^ The HF, MP2-F12, CCSD­(T), and CCSD­(T)-F12-c calculations were carried out using Molpro,^45^ while the PBE0 and ωB97M-V calculations were carried out using Gaussian 16 ^46^ and Q-Chem,^47^ respectively. SD-DMC calculations with both HF and PBE0 orbitals were considered, with the orbitals being generated using PySCF.^48^ The DMC calculations were carried out using the QMCPACK^49,50^ program. For all eight molecules considered, the CASSCF procedure was used to generate trial wave functions for the MD-DMC calculations, with the active spaces described below. The CASSCF calculations were carried out with GAMESS.^51^ For the PQ/HQ pair, we also report in the SI the results of energy differences from configuration interaction using perturbative selection done iteratively (CIPSI)^52^ variant of the selected configuration interaction (SCI) method as well as MD-DMC calculations using CIPSI trial wave functions.

The energy differences for the quinone/nonquinone pairs are reported in Table 1, while the relative energies of the three benzoquinone isomers are reported in Figure 2. For the PQ/HQ pair of molecules the CCSD­(T)-F12-c energy differences obtained using the aug-cc-pVTZ and aug-cc-pvQZ basis sets^39,40^ agree to within 0.4 kcal/mol leading us to conclude that the CCSD­(T)-F12-c/aug-cc-pVQZ result is close to the CBS limit. For the N protomers of 4ABA the CCSD­(T)-F12-c calculations using the aug-cc-pVDZ and aug-cc-pVTZ basis sets give energy differences that agree to within 0.1 kcal/mol. This indicates that for these species the CCSD­(T)-F12-c energy difference is well converged when using the aug-cc-pVTZ basis set. In addition, with the cc-pVTZ basis set, the CCSD­(T) procedure (without F12) gives an energy difference for PQ and HQ very close to that of approximate full configuration interaction (FCI) calculations (see the SI), justifying the use of the CCSD­(T)-F12-c calculations for providing the benchmark reference values.

**Table 1:** PQ – HQ, *p*-Xylylene – *p*-Xylene and O – N 4ABA Protomer Energy Differences at Various Levels of Theory^a^

Method^a^	*E*(PQ) – *E*(HQ)	*E*(*p*-xylylene) – *E*(*p*-xylene)	*E*(O) – *E*(N)
PBE0/aug-cc-pVQZ	782.4	794.9	–9.8
ωB97M-V/aug-cc-pVQZ	775.2	790.5	–6.5
HF/aug-cc-pVQZ	749.7	769.3	–8.4
MP2-F12-c/aug-cc-pVQZ	780.6	794.0	–1.7
CCSD(T)/cc-pVDZ	769.6	786.4	–0.1
CCSD(T)/cc-pVTZ	775.7	791.3	–3.2
CCSD(T)/cc-pCVTZ	776.1		–3.3
CCSD(T)-F12-c/aug-cc-pVDZ	776.6	790.5	–3.7
CCSD(T)-F12-c/aug-cc-pVTZ	778.3	792.4	–3.5
CCSD(T)-F12-c/aug-cc-pVQZ	778.7		
SD-DMC/cc-pVTZ (HF)	783.7	797.7	–3.6
SD-DMC/cc-pVXZ (HF)^b^	784.8		–4.2
SD-DMC/cc-pVTZ (PBE0)	782.5	796.4	–5.3
SD-DMC/cc-pVXZ (PBE0)^b^	783.2		–5.4
CAS-DMC/cc-pVTZ	779.4	791.9	–4.7

a Energies are given in kcal/mol.

b The orbitals used for the SD-DMC are indicated in parentheses.

c For the SD-DMC calculations, results obtained using the cc-pV5Z basis set are reported for PQ/HQ, with the cc-pVQZ basis set for the 4ABA protomers and with the cc-pVTZ basis set for the *p*-xylylene – *p-*xylene energy difference.

**Figure 2:**
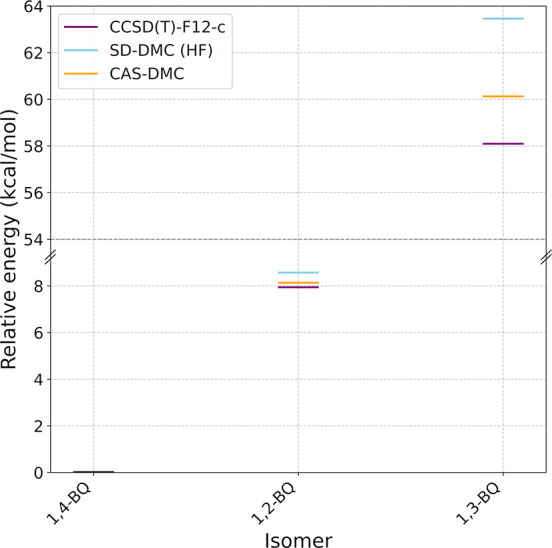
Energies of 1,2-benzoquinone and 1,3-benzoquinone relative to 1,4-benzoquinone, computed using the CCSD­(T)-F12-c, SD-DMC (HF), and CAS-DMC methods. The DMC calculations used the cc-pVTZ basis set, while CCSD­(T)-F12-c calculations were carried out using the aug-cc-pVTZ basis set.

For PQ and HQ SD-DMC calculations were carried out using the cc-pVTZ, cc-pVQZ, and cc-pV5Z basis sets,^39^ and the SD-DMC calculations for the two 4ABA protomers were carried out using the cc-pVTZ and cc-pVQZ basis sets. For the other four species considered, only the cc-pVTZ basis set was used in the SD-DMC calculations. The MD-DMC calculations carried out using CASSCF trial wave functions hereafter will be termed CAS-DMC. The CAS wave functions employed the cc-pVTZ basis set and included all valence orbitals of π symmetry. This resulted in CAS­(8,8) for the three benzoquinone isomers, *p*-xylylene and *p*-xylene; CAS­(10,8) for HQ; and CAS­(12,10) for the 4ABA protomers. The orbitals for these CAS spaces are shown in the SI.

To reduce equilibration times and to accelerate the convergence of both SD and MD-DMC calculations, the trial wave functions included Jastrow factors^53,54^ comprised of one-body, two-body, and three-body Jastrow terms, with 10, 10, and 26 parameters, respectively. The Jastrow parameters were optimized using the variational Monte Carlo (VMC) procedure (with a 95% weight on energy minimization and a 5% weight on variance minimization). CAS-DMC calculations were carried out with the CI coefficients from the CASSCF calculations as well as with the coefficients optimized together with the Jastrow parameters. The main manuscript reports energy differences obtained from the CAS-DMC calculations with coefficient reoptimization, while the results without coefficient reoptimization are reported in the SI. All DMC calculations were performed with a time step of 0.001 au, which is sufficiently small to obviate the need for time step extrapolation. We used 30 steps to minimize autocorrelation and 32,000 walkers to reduce population bias. For SD-DMC and CAS-DMC, the number of blocks was adjusted for each system to achieve error bars of 0.3 kcal/mol.

As seen from Table 1, the CCSD­(T)-F12-c/aug-cc-pVQZ calculations give a PQ – HQ energy difference of 778.7 kcal/mol, much larger than the HF/aug-cc-pVQZ energy difference of 749.7 kcal/mol. While these results indicate a significantly larger correlation correction for HQ than for PQ, on a per valence electron basis the correlation correction is larger in PQ than in HQ. The PBE0 and ωB87M-V values of the PQ – HQ energy difference are 782.4 and 775.2 kcal/mol, respectively, with these values differing by 3.5–3.7 kcal/mol in magnitude from the CCSD­(T)-F12-c/aug-cc-pVQZ result. Table 1 reports the SD-DMC differences for the largest basis set used for each pair of molecules, with results for the smaller basis sets being reported in the SI. The SD-DMC PQ – HQ energy difference is found to be relatively insensitive to the orbitals used (PBE0 or HF) and to the basis set employed. With the cc-pV5Z basis set, the SD-DMC PQ – HQ energy differences are 784.8 kcal/mol (HF) and 783.2 kcal/mol (PBE0). These values are, respectively, 6.1 and 4.5 kcal/mol larger than the CCSD­(T)-F12-c/aug-cc-pVQZ value of the energy difference. This is consistent with a greater nodal surface error for PQ than for HQ. Most noteworthy is the finding that the CAS-DMC/cc-pVTZ calculations give a PQ – HQ energy difference of 779.4 kcal/mol, within 0.7 kcal/mol of the CCSD­(T)-F12-c result. We note also that, with the smaller cc-pVTZ basis set, the SD-DMC value of the PQ – HQ energy difference differs by about 1 kcal/mol from the value obtained using the larger cc-pV5Z basis set. Thus, for the systems for which only the smaller cc-pVTZ basis set was used for the DMC calculation, the errors in the energy differences due to basis set limitations could be on the order of 1 kcal/mol.

While the coupled cluster calculations correlate only the valence electrons, the DMC calculations correlate all of the electrons in each species. To check the importance of core–valence correlation on the relative energies, we calculated the CCSD­(T) energies of PQ and HQ using the cc-pVTZ basis set and the frozen-core approximation as well as with the cc-pCVTZ basis set^55^ and correlating all electrons. Correlation effects involving the core electrons change the HQ – PQ energy difference by only 0.4 kcal/mol.

For *p*-xylene and *p*-xylylene the HF calculations predict an energy difference of 769.3 kcal/mol as compared with the 792.4 kcal/mol value of the energy difference from the CCSD­(T)-F12-c/aug-cc-pVTZ calculations. The corresponding PBE0 and ωB97M-V results are 794.9 and 790.5 kcal/mol, respectively, differing by 2.5–1.9 kcal/mol from the CCSD­(T)-F12-c/aug-c-pVTZ reference. As for the HQ – PQ energy difference, SD-DMC calculations predict significantly larger values of the *p*-xylylene – *p*-xylene energy difference, with the SD-DMC/cc-pVTZ (HF) and SD-DMC-ccpVTZ (PBE0) differences being 797.4 and 796.4 kcal/mol, respectively. In contrast, CAS-DMC/cc-pVTZ calculations give an energy difference of 791.9 kcal/mol, within 0.5 kcal/mol of the CCSD­(T)-F12-c/aug-cc-pVTZ reference, highlighting that CAS-DMC accurately reproduces the benchmark value.

For 4ABA the HF, PBE0 and ωB97M-V calculations (all with the aug-cc-pvQZ basis set) place the O-protomer below the N-protomer by 8.4, 9.7, and 6.5 kcal/mol, respectively, while the CCSD­(T)-F12-c/aug-cc-pVTZ, SD-DMC/cc-pvQZ­(HF) and SD-DMC/cc-pVQZ­(PBE0) calculations place the O protomer energetically below the N protomer by 3.5, 4.2, and 5.4 kcal/mol, respectively. It is seen from these results that electron correlation effects are less important in establishing the energy difference between the two 4ABA protomers than for the energy differences between PQ and HQ and between *p*-xylylene and *p*-xylene. In addition, SD-DMC calculations (both using HF and PBE0 orbitals) give energy differences between the 4ABA protomers in closer agreement with the CCSD­(T)-F12-c result than that found for the HQ – PQ and *p*-xylylene – *p*-xylene energy differences. The CAS­(12,10)-DMC calculations place the O protomer energetically 4.7 kcal/mol below the N protomer, an energy separation only 1.2 kcal/mol smaller than the CCSD­(T)-F12-c/aug-cc-pVQZ result. Thus, in this case, the adoption of the π-space CAS trial wave functions increased the DMC energy of the aromatic isomer more than that of the quinoidal isomer.

The relative energies of the three benzoquinone isomers are listed in Figure 2. The energy difference between the 1,2- and 1,4-benzoquinone isomers is calculated to be 7.9, 8.6, and 8.1 kcal/mol with the CCSD­(T)-F12-c, SD-DMC (HF), and CAS-DMC methods, respectively. In this case both the SD-DMC and CAS-DMC calculations give values of the energy difference in good agreement with the CCSD­(T)-F12-c result. In contrast, the energy difference between the 1,3- and 1,4-benzoquinone isomers is calculated to be 58.1, 63.5, and 60.1 kcal/mol with the CCSD­(T)-F12-c, SD-DMC (HF) and CAS-DMC methods, respectively. These results are consistent with 1,3-benzoquinone having the greatest diradical character (see CAS coefficients in the SI). The 2 kcal/mol discrepancy between the CCSD­(T)-F12-c and CAS-DMC values of the energy difference between the 1,3- and 1,4-benzoquinone systems could reflect the inadequacy of CCSD­(T)-F12-c for 1,3-benzoquinone due to its high diradical character.

The coefficients of the dominant configurations of the CAS wave functions, before and after optimization in the VMC step, are listed in the SI. The trends in the vectors for the CASSCF calculations are consistent with those in the energy differences discussed above. For example, the leading configurations in CASSCF calculations on PQ and HQ after optimization in the VMC step are 0.958 and 0.976, respectively. In addition, the CAS wave function of PQ (after coefficient optimization in VMC) has nine configurations with coefficients larger than 0.05 in magnitude, while the corresponding CAS wave function of HQ has only five configurations with coefficients larger than 0.05 in magnitude. These results are consistent with stronger static correlation in PQ than HQ. Similar trends are observed for *p*-xylylene and *p*-xylene. Namely, the leading coefficients are 0.953 for *p*-xylylene and 0.979 for *p*-xylene, respectively. In addition, *p*-xylylene has 11 configurations with coefficients larger than 0.05 in magnitude, whereas *p*-xylene has only seven, indicating a greater static correlation in the former.

For the O and N protomers of 4ABA, the leading coefficients in the CAS wave functions after coefficient optimization in the VMC step are 0.973 and 0.965, respectively. Consequently, SD-DMC is more successful in predicting the energy difference between the two 4ABA protomers than the difference between HQ and PQ.

Among the three benzoquinone isomers, 1,3-benzoquinone exhibits the lowest leading CAS coefficient (after reoptimization) (0.873), compared to 1,2-benzoquinone (0.959) and PQ (0.958). The stronger static correlation in 1,3-benzoquinone relative to the other isomers aligns with the relative energy differences presented in Figure 2. This is further supported by the number of configurations with coefficients above 0.05 in magnitude—13 for 1,3-benzoquinone—indicating stronger multiconfigurational character. Accordingly, SD-DMC yields the largest deviation from the CCSD­(T)-F12-c reference for this isomer, highlighting the method’s reduced accuracy as diradical character increases.

For five of the eight species considered, the DMC energies obtained with CAS trial wave functions without coefficient reoptimization in VMC are higher in energy than those obtained from the SD-DMC (HF) calculations using the same basis set. Moreover, the energy differences obtained from DMC calculations using these CAS trial wave functions are in poorer agreement with the CCSD­(T)-F12-c results than the energy differences from SD-DMC calculations. The fact that the DMC energy can increase upon adding additional Slater determinants to the trial wave function has been noted previously.^56–58^ When the CAS coefficients are reoptimized in the presence of the Jastrow factor, the weights of the most excited configurations decrease in magnitude, and the subsequent CAS-DMC calculations now give energies below the SD-DMC energies and energy differences that are close to the CCSD­(T)-F12-c results. Thus, reoptimizing the CAS coefficients along with the parameters of the Jastrow functions is crucial for accurately capturing the static correlation that is important for describing the nodal surfaces for the extended conjugated systems considered here.

In this study, we examined the performance of various electronic structure methods for predicting the energy differences between three pairs of related molecules with quinoidal and aromatic character as well as between different quinoidal isomers. Of special interest is the performance of the SD-DMC and MD-DMC calculations. The error in the SD-DMC value of the energy difference is found to be greater for the HQ/PQ pair and the *p*-xylylene/*p*-xylene pair than for the two protomers of 4ABA. This is consistent with the greater role of static correlation in PQ and *p*-xylylene than in the O-protomer of 4ABA. A key result is that DMC calculations using CASSCF trial wave functions in which the CI coefficients are reoptimized together with the Jastrow parameters yield energy differences between the quinoidal and associated aromatic pair in close agreement (∼1 kcal/mol) with the CCSD­(T)-F12-c results. In contrast, CAS-DMC without coefficient reoptimization can give higher energies than obtained from the SD-DMC (HF) calculations and energy differences that differ considerably from the CCSD­(T)-F12-c benchmark results. Examination of the CAS coefficients reveals that weights of the most important excited configurations are considerably reduced when they are reoptimized together with the Jastrow parameters in a VMC step. We speculate that this is because the CAS wave functions recover both dynamic and static correlation, which can lead to significantly larger weights for the excited configurations than found when the CAS coefficients were reoptimized together with the Jastrow factor.

Geometries and HF, DFT, SCI, CCSD­(T)-F12-c, MP2 and DMC energies for the molecules considered are available at the Materials Data Facility (10.18126/13XF-0NXN).^59,60^

## Supplementary Material



## Notes

Optimized geometries of the eight systems studied computed at the MP2/aug-cc-pVDZ level of theory; CIPSI results for PQ and HQ systems, including total energies; computational details of MD-DMC calculations using CIPSI trial wave functions for PQ and HQ systems; SD-DMC energies using cc-pVTZ, cc-pVQZ and cc-pV5Z basis sets for HQ/PQ and with the cc-pvTZ and cc-pVQZ basis sets for the 4ABA protomers alongside the CCSD­(T)-F12-c energies; VMC and DMC energies using HF or CASSCF orbitals with the cc-pVTZ basis set; CASSCF results showing the 10 most important configurations for the eight studied species, along with their corresponding CAS spaces (PDF)

## §

Chembricks, Inc, 1-469 Waverley St., Menlo Park, CA 94025, United States
