# Supplementary material for: Performance of Diffusion Monte Carlo Calculations for Predicting the Relative Energies of Quinoidal and Nonquinoidal Species
Source: J Phys Chem Lett. 2025 Dec 12;16(51):13094–100. doi: 10.1021/acs.jpclett.5c03120 (PMC12746463; doi:10.1021/acs.jpclett.5c03120)
Supplement: Supplementary file 1 [file jz5c03120_si_001.pdf]

Supporting Information for

Performance of Diffusion Monte Carlo Calculations for Predicting the Relative Energies of Quinoidal and Non-quinoidal Species

N. Mauger,[1] A. Benali,[2,†] and K. D. Jordan[1*]
nastasia.mauger@pitt.edu, abenali.sci@gmail.com, jordan@pitt.edu

[1]Department of Chemistry, University of Pittsburgh, Pittsburgh, PA 15218, United States

[2]Computational Science Division, Argonne National Laboratory, Lemont, IL 60439, United States

Paraquinone geometry (Å) optimized at the MP2/aug-cc-pVDZ level of theory

C 1.448059 0.000000 0.000000
C -1.448059 0.000000 0.000000

C	0.680318	-1.274107	0.000000
C	0.680318	1.274107	0.000000
C	-0.680318	1.274107	0.000000
C	-0.680318	-1.274107	0.000000
O	2.688591	0.000000	0.000000
O	-2.688591	0.000000	0.000000
H	1.267706	-2.197259	0.000000
H	1.267706	2.197259	0.000000
H	-1.267706	2.197259	0.000000
H	-1.267706	-2.197259	0.000000

Hydroquinone geometry (Å) optimized at the MP2/aug-cc-pVDZ level of theory

C	1.219900	-0.702569	0.000000
O	2.451626	-1.333872	0.000000
H	2.306497	-2.291021	0.000000
C	1.219441	0.702122	0.000000
C	0.000534	1.402927	0.000000
C	-1.219900	0.702569	0.000000
O	-2.451626	1.333872	0.000000
H	-2.306497	2.291021	0.000000
C	-1.219441	-0.702122	0.000000
C	-0.000534	-1.402927	0.000000
H	-0.006758	-2.498629	0.000000
H	-2.172812	-1.236846	0.000000
H	0.006758	2.498629	0.000000
H	2.172812	1.236846	0.000000

p–xylylene (Å) optimized at the MP2/aug-cc-pVDZ level of theory

C	1.264928	-0.707707	0.000000
C	2.460890	-1.377037	0.000000
H	2.495420	-2.469804	-0.000000
H	3.409493	-0.832834	0.000000
C	1.205712	0.754314	0.000000
C	0.012191	1.422070	0.000000
C	-1.264928	0.707707	0.000000
C	-2.460890	1.377037	0.000000
H	-2.495420	2.469804	-0.000000
H	-3.409493	0.832834	0.000000
C	-1.205712	-0.754314	0.000000
C	-0.012191	-1.422070	0.000000
H	0.006850	-2.517418	-0.000000
H	-2.149230	-1.311019	0.000000
H	-0.006850	2.517418	-0.000000

H 2.149230 1.311019 0.000000

p–xylene geometry (Å) optimized at the MP2/aug-cc-pVDZ level of theory

C 0.108252 -0.060565 0.145359
C -1.212316 0.678271 0.111708
C -1.257016 2.088167 0.107580
C -2.484731 2.775053 0.107580
C -3.709642 2.075485 0.111708
C -5.030210 2.814321 0.145359
H -4.950335 3.792213 -0.354664
H -5.821787 2.234614 -0.354664
H -5.351505 2.994080 1.185266
C -3.664942 0.665589 0.107580
C -2.437227 -0.021298 0.107580
H -2.431517 -1.116947 0.095917
H -4.601651 0.097208 0.095917
H -2.490441 3.870703 0.095917
H -0.320308 2.656548 0.095917
H 0.028377 -1.038457 -0.354664
H 0.899828 0.519141 -0.354664
H 0.429547 -0.240324 1.185266

1,2–benzoquinone (Å) optimized at the MP2/aug-cc-pVDZ level of theory

C -0.014053 -0.026347 0.000000
O -1.083658 -0.640210 0.000000
C -0.013940 1.533441 0.000000
O -1.083455 2.147460 0.000000
C 1.297716 2.216353 0.000000
C 2.452957 1.488053 0.000000
C 2.452850 0.018683 0.000000
C 1.297503 -0.709449 0.000000
H 1.295075 -1.803191 0.000000
H 3.418971 -0.495574 0.000000
H 3.419152 2.002170 0.000000
H 1.295446 3.310096 0.000000

1,3–benzoquinone (Å) optimized at the MP2/aug-cc-pVDZ level of theory

C 0.017930 0.078391 0.000000
O 1.200539 0.555281 0.000000
C -1.209241 0.784453 0.000000
C -2.434733 0.075481 0.000000
O -3.618471 0.549563 0.000000

C	-2.387650	-1.413594	0.000000
C	-1.205747	-2.159922	0.000000
C	-0.025620	-1.410791	0.000000
H	0.957782	-1.897234	0.000000
H	-1.204451	-3.252174	0.000000
H	-3.369895	-1.902369	0.000000
H	-1.210537	1.876173	0.000000

O–protomer of 4ABA geometry (Å) optimized at the MP2/aug-cc-pVDZ level of theory

C	-0.655574	0.012816	0.000293
C	0.056353	-1.224493	-0.000046
C	1.441072	-1.232477	-0.000230
C	2.175060	-0.006708	-0.000079
C	1.459221	1.230277	0.000606
C	0.073339	1.238080	0.000696
C	-2.074878	-0.005270	-0.000012
H	-0.492226	-2.169005	-0.000292
H	1.980908	-2.183624	-0.000617
N	3.525793	-0.015180	-0.000325
H	2.011032	2.174331	0.001054
H	-0.427316	2.212507	0.001515
O	-2.711297	-1.152931	0.000703
O	-2.870858	1.047851	-0.001205
H	4.057757	0.846731	-0.000705
H	4.044781	-0.885093	-0.001178
H	-2.368614	1.881425	-0.001411
H	-3.677190	-1.003723	0.000568

N–protomer of 4ABA geometry (Å) optimized at the MP2/aug-cc-pVDZ level of theory

C	-0.737010	-0.027040	0.000030
C	-0.061070	1.209560	0.000010
C	1.344820	1.236650	-0.000010
C	2.025200	0.013300	-0.000050
C	1.381750	-1.232160	-0.000110
C	-0.021110	-1.242280	-0.000080
C	-2.239690	-0.111630	0.000080
H	-0.626510	2.143380	0.000030
H	1.881430	2.191230	0.000020

N	3.517180	0.031110	0.000080
H	1.946920	-2.169980	-0.000180
H	-0.575450	-2.184180	-0.000140
O	-2.811100	1.117710	-0.000090
O	-2.851310	-1.164810	0.000120
H	3.900550	-0.438360	0.832210
H	3.871150	0.996940	-0.000490
H	3.900730	-0.439410	-0.831380
H	-3.777080	0.981130	-0.000240

CIPSI calculations

In the initial CIPSI calculations on PQ and HQ using the cc-pVTZ basis set, the largest expansions included approximately 1.6 million and 1.7 million Slater determinants. The corresponding variances were 2.7 and 3.5 Ha^2 for PQ and HQ, respectively. These results indicate that, despite the large determinant expansions, the SCI calculations are far from converged. While running even larger SCI calculations is computationally feasible, MD-DMC calculations with the resulting trial wave functions would be intractable. To compact the size of the determinant spaces we adopted an iterative generation of natural orbitals (NOs) with the process being repeated until convergence of the E+rPT2 energy was achieved with a fixed number (~3.5 million) of determinants. This required four iterations for both HQ and PQ. The CIPSI calculations were carried out using the Quantum Package 2 (QP2) program.[1]

The major challenge here is that with the basis set used one is forced to truncate the SCI iterative procedure well before convergence. As a result, the energy differences as a function of the number of iterations can exhibit oscillatory behavior, making it difficult to extrapolate to the FCI limit. Achieving a well-converged PQ – HQ energy difference from such an extrapolation would require significantly larger SCI calculations. An alternative approach is to exploit the fact that the SCI energies are determined iteratively, with increasing size of the variational space and an evaluation of the renormalized second-order perturbation theory (rPT2) correction[2,3] at each iteration. This enables us to interpolate the total (variational

plus rPT2 correction) SCI energies of HQ and PQas well as their energy difference as a function of the rPT2 energy. This approach gives a stabilized energy difference at -775.3 kcal/mol, as shown in Figure S1. This result is in close agreement with the -775.7 kcal/mol value of the energy difference obtained from CCSD(T)/cc-pVTZ calculations using the same basis set (i.e., cc-pVTZ). Although this demonstrates the value of using interpolation of rPT2 values as a way of estimating energy differences in SCI calculations, we note that there is about a 3 kcal/mol error in this energy difference compared to that from the CCSD(T)-F12-c/aug-cc-pVQZ calculations. However, it would be computationally prohibitive to extend these calculations to the significantly larger basis sets needed to obtain an energy difference in close agreement with the CCSD(T)-F12-c/aug-cc-pVTZ calculations

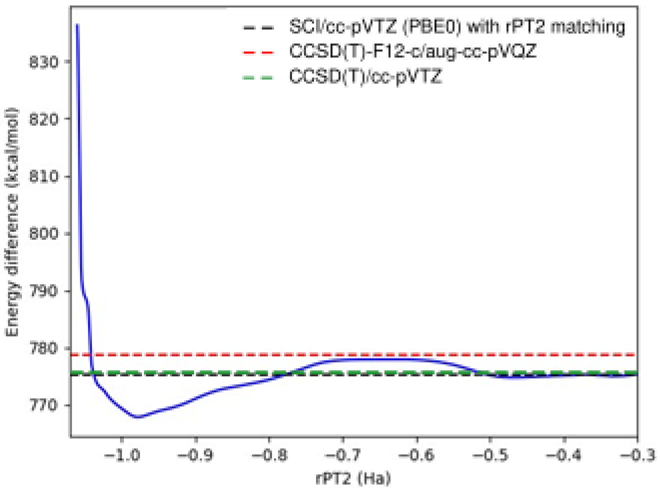

Fig. S1: Difference in the SCI energies of PQ and HQ as a function of the rPT2 correction.

<u>MD-DMC using CIPSI trial wave functions</u>

MD-DMC calculations were performed using SCI expanions that included all Slater determinants with coefficients larger than 10^{-2}, 10^{-3}, and 10^{-4} in magnitude. Determinants with smaller coefficients were

excluded.The protocol for these MD-DMC calculations was the same as that for the SD-DMC calculations, except for the number of blocks. Specifically, 3000, 900, and 100 blocks were used for the 10^{-2}, 10^{-3}, and 10^{-4} coefficient cutoffs in the PQ and HQ calculations, yielding statistical errors of 0.4 kcal/mol for the 10^{-2} and 10^{-3} cutoffs and 0.6 kcal/mol for the 10^{-4} case. The resulting wave function expansions contained 58, 27,336, and 980,795 determinants for HQ and 104, 26,790, and 822,606 for PQ. The corresponding PQ – HQ energy differences were 777.6, 774.8, and 776.1 kcal/mol, which are, respectively, 1.1, 3.9, and 2.6 kcal/mol lower than the CCSD(T)-F12-c/aug-cc-pVQZ result. It is clear from these results that using a truncation at a fixed coefficient size in the SCI calculations does not lead to a balanced treatment of the nodal surface error in the two molecules. However, the fact that the MD-DMC calculations with the largest coefficient cutoff give an energy difference closest to the CCSD(T)-F12-c result may not be fortuitous. The excited Slater determinants that enter the SCI wave function with the largest magnitude coefficients tend to be important for describing static correlation, which can significantly impact the nodal surface. The Slater determinants that enter the SCI wave function with small coefficients tend to be more important for dynamic correlation, and these are especially hard to treat in a balanced manner in calculating energies differences. Ideas along this line have been expressed by Yuan, Chang, and Wagner.[4]

SD-DMC

Table S1. CCSD(T)-F12-c/aug-cc-pVQZ and SD-DMC energies (a.u.) using different basis set for paraquinone and hydroquinone systems.

	Paraquinone	Hydroquinone
CCSD(T)-F12-c/aug-cc-pVQZ	-380.98659573	-382.22754969
SD-DMC/cc-pVTZ (HF)	-381.350431 ± 0.000421	-382.599362 ± 0.000358
SD-DMC/cc-pVQZ (HF)	-381.35577 ± 0.000401	-382.604682 ± 0.000485
SD-DMC/cc-pV5Z (HF)	-381.356097 ± 0.000436	-382.606746 ± 0.000392

SD-DMC/cc-pVTZ (PBE0)	-381.36533 ± 0.000614	-382.612374 ± 0.00052
SD-DMC/cc-pVQZ (PBE0)	-381.369992 ± 0.00037	-382.616252 ± 0.00042
SD-DMC/cc-pV5Z (PBE0)	-381.371044 ± 0.0004	-382.619256 ± 0.000426

[a]The orbitals used for the SD-DMC are indicated in parentheses.

Table S2. CCSD(T)-F12-c/aug-cc-pVTZ and SD-DMC energies (a.u.) using different basis set for 4ABA protomers

	O–protomer	N–protomer
CCSD(T)-F12-x/aug-cc-pVTZ	-475.91656184	-475.91092064
SD-DMC/cc-pVTZ (HF)	-476.420778 ± 0.000442	-476.414997 ± 0.000352
SD-DMC/cc-pVQZ (HF)	-476.425778 ± 0.000510	-476.419023 ± 0.000424
SD-DMC/cc-pVTZ (PBE0)	-476.438599 ± 0.000389	-476.430235 ± 0.000444
SD-DMC/cc-pVQZ (PBE0)	-476.444074 ± 0.000432	-476.435484 ± 0.000372

[a]The orbitals used for the SD-DMC are indicated in parentheses.

<u>DMC energies</u>

Table S3. VMC and DMC energies (a.u.) using HF and CASSCF trial wave functions, the latter with or without reoptimization of the CI coefficients in the VMC step.

	Paraquinone	Hydroquinone	O–protomer	N–protomer
SD-VMC (HF)	-381.115030 ± 0.003018	-382.367737 ± 0.000705	-476.132019 ± 0.000806	-476.123266 ± 0.000414
SD-DMC (HF)	-381.350431 ± 0.000421	-382.599362 ± 0.000358	-476.420778 ± 0.000442	-476.414997 ± 0.000352
CAS-VMC	-381.135307 ± 0.001059	-382.382219 ± 0.000858	-476.156829 ± 0.001472	-476.143076 ± 0.001279
CAS-DMC	-381.346982 ± 0.000371	-382.598277 ± 0.000386	-476.422716 ± 0.000367	-476.409243 ± 0.000386
CAS-VMC (coeff opt)	-381.153370 ± 0.000868	-382.393811 ± 0.001288	-476.165244 ± 0.001406	-476.157294 ± 0.001368

CAS-DMC (coeff opt)	-381.368532 ± 0.000415	-382.610523 ± 0.000373	-476.434936 ± 0.000367	-476.427420 ± 0.000410

Table S4. VMD and DMC energies (a.u.) using HF and CASSCF trial wave functions, the latter with or without reoptimization of the CI coefficients in the VMC step.

	p–xylylene	*p*–xylene	1,2–benzoquinone	1,3–benzoquinone
SD-VMC (HF)	-309.297080 ± 0.000972	-310.570209 ± 0.000597	-381.097114 ± 0.000886	-381.009173 ± 0.000776
SD-DMC (HF)	-309.522954 ± 0.000400	-310.794242 ± 0.000380	-381.336771 ± 0.000325	-381.249299 ± 0.000332
CAS-VMC	-309.324412 ± 0.000845	-310.582584 ± 0.000714	-381.118020 ± 0.000858	-381.038885 ± 0.000717
CAS-DMC	-309.526725 ± 0.000368	-310.792380 ± 0.000346	-381.334039 ± 0.000392	-381.253978 ± 0.000384
CAS-VMC (coeff opt)	-309.339944 ± 0.001057	-310.594563 ± 0.000695	-381.140166 ± 0.000790	-381.057623 ± 0.001872
CAS-DMC (coeff opt)	-309.542414 ± 0.000386	-310.804370 ± 0.000379	-381.355566 ± 0.000385	-381.272709 ± 0.000411

<u>CASSCF wave functions</u>

Paraquinone – CASSCF(8,8)			Hydroquinone – CASSCF(10,8)		
Configuration	Coefficient before optimization	Coefficient after optimization	Configuration	Coefficient before optimization	Coefficient after optimization
22220000	0.907633	0.957728	22222000	0.940262	0.976456
22022000	-0.147013	-0.123218	22211110	0.132967	0.09806
22111100	0.158969	0.120727	22202200	-0.148126	-0.097575
21121010	0.127374	0.083554	22220020	-0.136304	-0.087524

22200200	-0.111155	-0.082686	22211110	-0.112239	-0.060434
11221010	-0.106149	-0.064213	22112101	-0.099283	-0.058968
22202000	-0.069467	-0.054822	22121011	-0.086069	-0.049251
12121001	-0.086359	-0.05359	22220200	-0.045442	-0.043263
12211100	0.070818	0.052096	12221011	-0.058546	-0.029794
22110101	0.076973	0.049394	22022200	-0.036329	-0.025676

[a]Singly and doubly occupied orbitals are denoted by 1 and 2, respectively.

Table S6: Dominant configurations in the CASSCF wave functions of the O and N–protomers both before and after optimization in the VMC step, with the configurations ordered (after optimization) from highest to lowest in magnitude. In specifying the configurations, the orbital orderings are those shown in Figure S2.

O–protomer – CASSCF(12,10)			N–protomer – CASSCF(12,10)		
Configuration	Coefficient before optimization	Coefficient after optimization	Configuration	Coefficient before optimization	Coefficient after optimization
2222220000	0.946718	0.973342	2222220000	0.923288	0.965237
2222020200	-0.122794	-0.086579	2222200200	-0.145096	-0.106498
2222202000	-0.087668	-0.077425	2222022000	-0.137601	-0.103449
2221121100	-0.081882	-0.054515	2222111100	-0.128763	-0.100295
2222111100	0.061522	0.053908	2222111100	0.109517	0.077239
2222111100	-0.053304	-0.039040	2212210110	0.082247	0.051366
2222200020	-0.059637	-0.038888	2212121010	-0.070499	-0.044084
1222221000	-0.009736	-0.038804	2212121001	-0.065926	-0.041809
2222110110	-0.051004	-0.038544	2212210101	0.065384	0.039531

Table S5: Dominant configurations in the CASSCF wave functions for paraquinone and hydroquinone both before and after optimization in the VMC step, with the configurations ordered

(after optimization) from highest to lowest in magnitude. In specifying the configurations, the orbital orderings are those shown in Figure S2.

2222022000	-0.048230	-0.038504	2220220011	0.062035	0.035310

[a]Singly and doubly occupied orbitals are denoted by 1 and 2, respectively.

Table S7: Dominant configurations in the CASSCF wave functions for *p*–xylylene and *p*–xylene both before and after optimization in the VMC step, with the configurations ordered (after optimization) from highest to lowest in magnitude. In specifying the configurations, the orbital orderings are those shown in Figure S3.

p–xylylene – CASSCF(8,8)			*p*–xylene – CASSCF(10,8)		
Configuration	Coefficient before optimization	Coefficient after optimization	Configuration	Coefficient before optimization	Coefficient after optimization
22220000	0.899362	0.953338	22222000	0.939146	0.979086
22202000	-0.210044	-0.179152	22202200	-0.148732	-0.091455
21211010	-0.163679	-0.107594	22220020	-0.145034	-0.088187
22111100	0.132623	0.091855	22211110	0.135955	0.087540
22020200	-0.113835	-0.076656	22211110	-0.113532	-0.058197
12121100	0.086539	0.056349	22112101	0.105260	0.057881
12211001	-0.082024	-0.045885	22121011	0.097205	0.054630
12120101	0.080084	0.044664	22220200	-0.036535	-0.036774
22110101	0.068523	0.043028	22202020	-0.030193	-0.029577
20220020	-0.075774	-0.042380	12221011	0.047500	0.024579

[a]Singly and doubly occupied orbitals are denoted by 1 and 2, respectively.

Table S8: Dominant configurations in the CASSCF wave functions of the 1,2 and 1,3–benzoquinone both before and after optimization in the VMC step, with the configurations ordered (after optimization) from highest to lowest in magnitude. In specifying the configurations, the orbital orderings are those shown in Figure S3.

1,2–benzoquinone – CASSCF(8,8)			1,3–benzoquinone – CASSCF(8,8)		
Configuration	Coefficient before optimization	Coefficient after optimization	Configuration	Coefficient before optimization	Coefficient after optimization
22220000	0.902753	0.958577	22220000	0.807099	0.873136
22202000	-0.147210	-0.125705	22202000	-0.293420	-0.293638
22111100	0.152996	0.108350	22112000	0.188825	0.164166
21211010	-0.101424	-0.071792	21221000	-0.202491	-0.153591
22022000	-0.077020	-0.061203	22201010	0.176238	0.153047
22201010	-0.079480	-0.057501	12212000	0.110007	0.080805
20222000	-0.073457	-0.052276	21211100	-0.099246	-0.074044
11221100	-0.081923	-0.051142	22111010	-0.095448	-0.071136
20221010	0.079566	0.049181	21211001	0.103526	0.067434
12121010	0.069039	0.043321	22022000	-0.069522	-0.062287

[a]Singly and doubly occupied orbitals are denoted by 1 and 2, respectively.

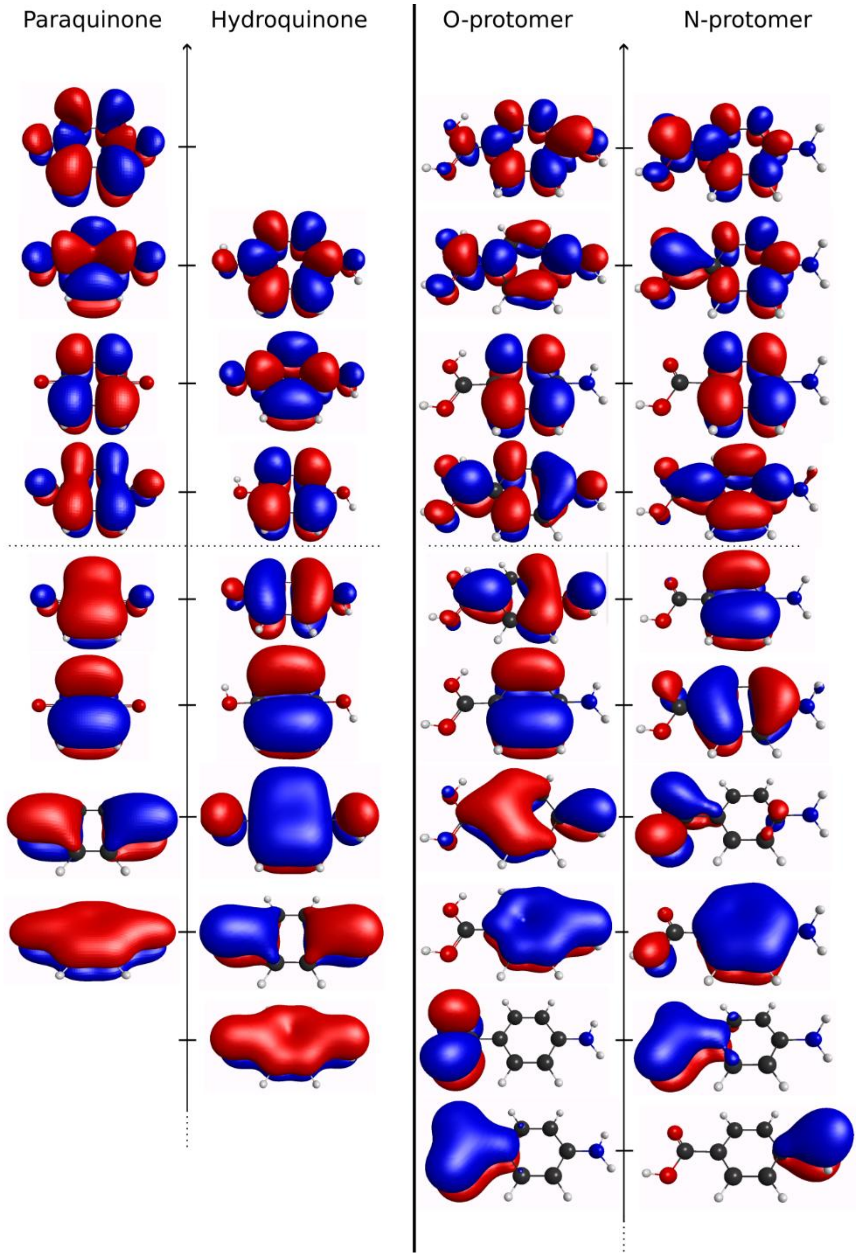

Fig. S2. Frontier π and π* orbitals of paraquinone, hydroquinone, and the O and N-protomers of 4ABA from CASSCF calculations.

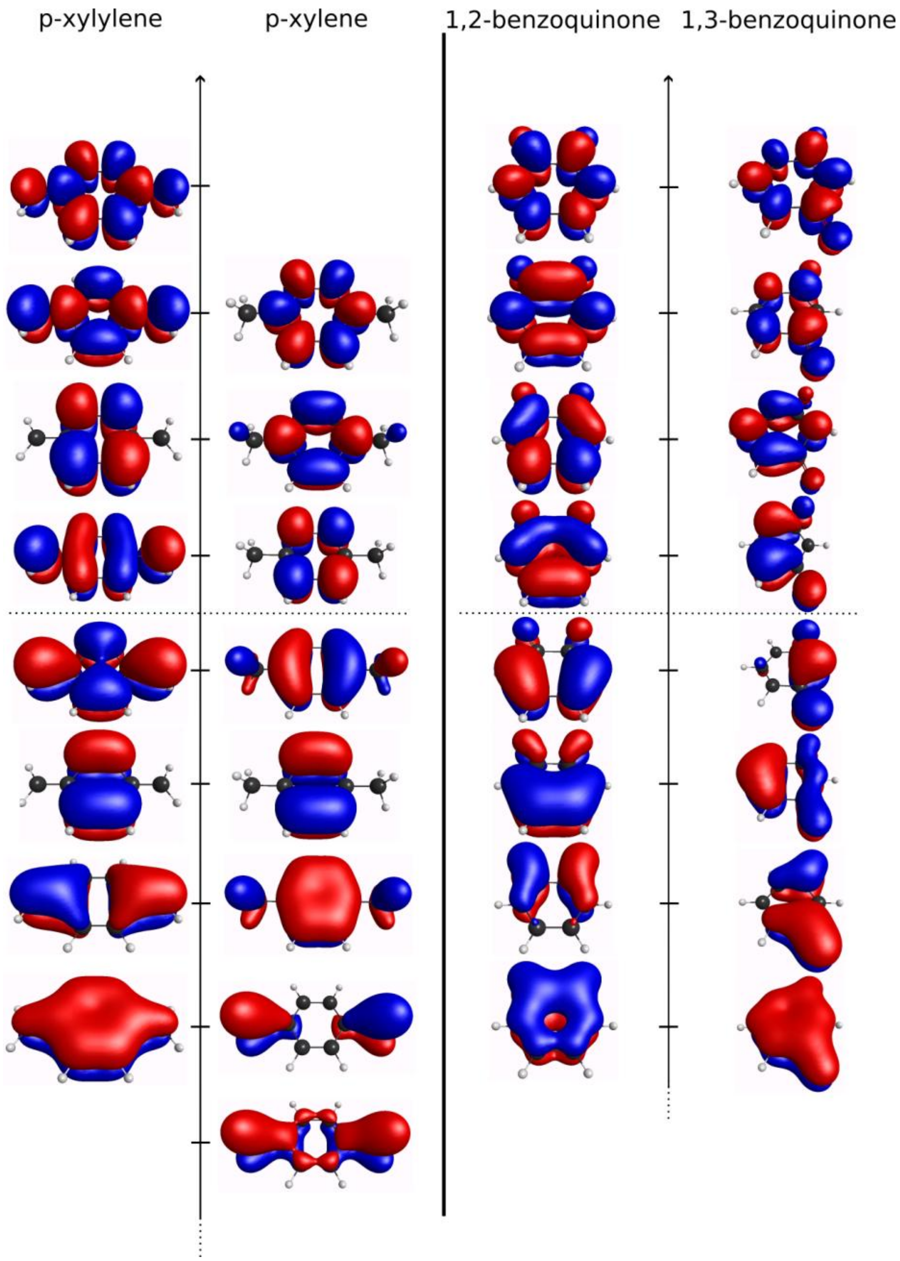

Fig. S3. Frontier π and π* orbitals of *p*–xylylene, *p*-xylene, 1,2–benzoquinone and 1,3–benzoquinone from CASSCF calculations.

References

1. Garniron, Y.; Applencourt, T.; Gasperich, K.; Benali, A.; Ferté, A.; Paquier, J.; Pradines, B.; Assaraf, R.; Reinhardt, P.; Toulouse, J.; et al.; Quantum Package 2.0: An open-source determinant-driven suite of programs. *J. Chem. Theory Comput*. **2019**, *15*, 3591–3609.

2. Heully, J. L.; Malrieu, J. P.; Zaitsevskii, A.; On the origin of size inconsistency of the second-order state-specific effective Hamiltonian method. *J. Chem. Phys*. **1996**, *105*, 6887–6891.

3. Garniron, Y.; Scemama, A.; Loos, P. F.; Caffarel, M.; Hybrid stochastic-deterministic calculation of the second-order perturbative contribution of multireference perturbation theory. *J. Chem. Phys*. **2017**, *147*, 034101.

4. Yuan, S.; Chang, Y.; Wagner, L. K.; Quantification of electron correlation for approximate quantum calculations. *J. Chem. Phys*. **2022**, *157*, 194101.
